# Functional conductivity imaging: quantitative mapping of brain activity

**DOI:** 10.1007/s13246-024-01484-z

**Published:** 2024-09-11

**Authors:** Jun Cao, Iain K. Ball, Benjamin Cassidy, Caroline D. Rae

**Affiliations:** 1 Neuroscience Research Australia, 139 Barker St, Randwick, NSW 2031 Australia; 2 Philips Australia & New Zealand, North Ryde, NSW 2113 Australia; 3 Pathfinder Exploration LLC, Tonopah, NV USA; 4 School of Psychology, The University of New South Wales, Sydney, NSW 2052 Australia

**Keywords:** Tissue conductivity, MREPT, bFFE, Visual stimulus, Somatosensory cortex

## Abstract

Theory and modelling suggest that detection of neuronal activity may be feasible using phase sensitive MRI methods. Successful detection of neuronal activity both in vitro and in vivo has been described while others have reported negative results. Magnetic resonance electrical properties tomography may be a route by which signal changes can be identified. Here, we report successful and repeatable detection at 3 Tesla of human brain activation in response to visual and somatosensory stimuli using a functional version of tissue conductivity imaging (funCI). This detects activation in both white and grey matter with apparent tissue conductivity changes of 0.1 S/m (17–20%, depending on the tissue baseline conductivity measure) allowing visualization of complete system circuitry. The degree of activation scales with the degree of the stimulus (duration or contrast). The conductivity response functions show a distinct timecourse from that of traditional fMRI haemodynamic (BOLD or Blood Oxygenation Level Dependent) response functions, peaking within milliseconds of stimulus cessation and returning to baseline within 3–4 s. We demonstrate the utility of the funCI approach by showing robust activation of the lateral somatosensory circuitry on stimulation of an index finger, on stimulation of a big toe or of noxious (heat) stimulation of the face as well as activation of visual circuitry on visual stimulation in up to five different individuals. The sensitivity and repeatability of this approach provides further evidence that magnetic resonance imaging approaches can detect brain activation beyond changes in blood supply.

**Supplementary Information:**

The online version contains supplementary material available at 10.1007/s13246-024-01484-z.

## Introduction

The fMRI workhorse, blood oxygenation level dependent (BOLD) fMRI relies on changes in magnetic susceptibility induced by altered blood flow and oxygenation state [1, 2]. The signal change detected is relative to a baseline level and is largely confined to well vascularized areas of brain [3]. It has been the mainstay of brain function research using MRI over the last thirty years since its inception. The holy grail of brain functional imaging has been to find a method that can detect neural activity with high spatial resolution across the whole brain in real time. This would enable better understanding of, and synergy with, the huge range of cognitive and functional processes with which the brain is engaged in daily life.

Much effort has been directed at finding a robust and reproducible method for magnetic resonance imaging of brain activation that can detect activation more immediate to neuronal activity than haemodynamic changes. These efforts range from detecting changes in the water diffusion signal [4, 5] to changes in the phase and magnitude of the MR signal arising from localized changes in the magnetic field due to neuronal currents (see below).

Modelling and work in phantoms has shown the possibility of detecting signal changes in the order of magnitude responding to the firing of neurons in response to stimuli [6–9]. An elegant experiment showed that activity generated by isolated organotypic rat brain tissue slices was sufficient to produce measurable changes in the phase of the magnetic field [10]. This experiment, in tissue independent of the circulatory system, also demonstrated that neuronal activity alone was sufficient to produce phase changes without any concomitant changes in blood flow. Successful detection of phase changes related to function in vivo has been reported (e.g. [11–15] but others have not detected any change [9, 16–18]. Recently, at 9.4 T, 2D line-scan-based gradient-echo method (DIANA) was used in mouse brain on a millisecond time scale [19] that showed high correlation with neural activity but others have reported inability to reproduce these results [20–22].

Separating the signal changes occurring due to neuronal firing from those due to blood flow changes has also presented challenges. Simultaneous recording of electrical and MR signals along with optogenetic stimulation in animal models may go some way in helping to unravel the source of the signal changes [23] although separating signal changes due to membrane potential alterations from changes due to electrical activity in brain may prove problematic since each relies on the other. Timing analysis of physiological responses to nerve activity suggest that the short term responses (< 1 s) are related to action potentials, while changes in cell volume [24] as well as blood oxygen or flow changes [25] occur on a longer (> 1 s) time scale.

Magnetic resonance electrical properties tomography (MREPT) is an approach that simultaneously maps tissue electrical properties, conductivity and permittivity, at the resonant frequency of the protons in water in a magnetic field [26–29]. The electric properties of tissues depend on their biophysical properties, such as ion concentration, water content, cell membrane capacitance, ion gated channels, and cellular structure. Action potentials in neurons generate ionic currents which in turn create changes in the local magnetic field and change tissue electrical properties. The electrical conductivity signal detected by MREPT may therefore be due to a combination of several physical processes.

Phase-MREPT is based on the transceive phase assumption [30, 31] that the phase of the positively-rotating component of ${\mathbf{B}}_{1}$ is half of the image’s transceive phase. In principle, the performance of phase-MREPT depends on the phase image quality. In a study [31] of phase-MREPT at 1.5T, 3T and 7T, the transceive phase assumption was found to be applicable at magnetic field strengths of 1.5T and 3T. Transceive phase is prone to unwanted phase contributions from off-resonance effects and eddy currents. Sequences based on refocusing pulses, such as spin-echo and turbo spin echo, are noted for exclusion of off-resonance effects. Another sequence frequently used for MREPT is the balanced fast field echo (bFFE). The high signal-to-noise ratio (SNR) with a short acquisition time, and the SNR of bFFE is dependent on pulse sequence efficiency [32], which implies that maximum available gradient strength and slew rates can be used to improve the SNR. Images acquired with bFFE may suffer from banding artefacts, which often occur at locations where the main field inhomogeneity is a multiple of 1/TR. Short-TR bFFE greatly reduces banding artifacts and preserves the quality of the resultant phase image. In this study, a short-TR bFFE (TR = 2.45 ms) sequence was used as it has recently been shown to be more precise and repeatable in brain than alternative approaches [33].

Advances in magnetic resonance technology and in the theory and assumptions behind the calculation of tissue conductivity [34] have meant that phase electrical properties tomography is now able to measure tissue conductivity with sufficient precision to be of utility. Recently, activation of the motor cortex was detected by magnetic resonance electrical properties tomography and reported in a conference abstract [35] where changes of 0.1 S/m were measured following clenching of fists or dorsiflexion of both feet. The authors scanned an axial slab of brain 28 mm deep covering mostly cortical (grey matter) areas with placement of the slab guided by previous BOLD imaging of the same task. The slab did not include white matter and a response function was not determined. The signal change was attributed to blood volume (BOLD) changes. Similarly, Schmidt [36] reported activation of the occipital cortex following a block design with 8 Hz flickering chequerboard presented for 60 s ON, 30 s OFF using SE-EPI which reported faster change in the phase of the signal from SE-EPI compared to the BOLD response. Conductivity values measured during visual stimulation show significant increases [37] which differs from the reported negative change in conductivity engendered by the BOLD response [38].

Here, we constructed an MR protocol and analysis approach which allows measurement of electrical tissue properties such as tissue conductivity across the entire brain on a spatial and temporal resolution which allows it to be used for functional conductivity imaging (funCI). We show the precision and repeatability of the method and the application of it to a range of stimuli designed to activate sensorimotor pathways as well as the visual pathway. We measure the response function to the stimuli and show that it closely follows the stimulus and is separate to the corresponding BOLD response.

## Methods

Scanning for this study was approved by the UNSW Human Research Ethics Committee (HC 190222) and by The University of Sydney (2020/737). Informed consent was obtained from all participants.

Visual and index finger stimulation funCI and BOLD visual stimulation data were acquired from four healthy males (median age 30, range 28–43 years) and one female (24 years). Data from funCI visual stimulation experiments used to calculate stimulus and contrast response functions were also obtained from healthy participants, one male 29 years and one female 24 years old. This latter acquisition was repeated on a separate date, same time of day, in both of these participants in order to determine response function repeatability. Data from funCI visual stimulation acquired at higher spatial resolution and BOLD finger scraping data was obtained from a healthy 21 years old female. Big toe stimulation funCI data was acquired from five control participants (one female (24 years) and four males (age range 31–38 years). Data from an acquisition using heat stimulation applied to the cheek was collected from one healthy male control (23 years).

### Image acquisition and experimental design

All images were acquired at 3 T using a 32 channel digital head coil (Ingenia CX, Philips, Best, The Netherlands). All imaging used two-channel parallel transmit SENSE [39].

${B}_{1}^{+}$ mapping used single-slice 2D DREAM [40]. T1-weighted structural images were acquired using 3D T1-turbo field echo (TR/TE/TI = 5.89/2.76/850 ms, resolution 1 $\times$ 1 $\times$ 1 ${\text{mm}}^{3}$, sagittal slices, FOV 240 $\times$ 240 × 189 ${\text{mm}}^{3}$, flip angle 8$^\circ$ compressed SENSE factor 4).

#### Visual stimuli

Visual stimulation funCI and BOLD visual stimulation data were acquired from four healthy males (median age 30, range 28–43 years) and one female (24 years). Visual stimuli were presented for 0.5 s as a flashing (8 Hz) greyscale (contrast 0.45) checkerboard at the beginning of the duty cycle, followed by 15 s of black screen. Tissue conductivity was estimated from images acquired using a 3D non-selective balanced fast field echo sequence (TR/TE 1.89/0.78 ms, 25° flip angle, compressed SENSE factor 4, 3 mm isotropic resolution, FOV = 240 × 240 × 189 mm^3^, sagittal acquisition, with dynamic scan time of 1.9 s and a total acquisition time of 7 min 14 s (220 dynamics (TRs) and 5 dummy scans). The robustness and repeatability of variants of the bFFE sequence used here and the rationale for ${B}_{1}^{+}$ mapping have been described previously [33]. BOLD (traditional fMRI) images were acquired for comparison purposes using the same stimulus and duty cycle, temporal and spatial resolution but with single-shot echo-planar imaging (TE = 30 ms, multiband = 2, SENSE = 3, 220 dynamics and 5 dummy scans).

To examine the effect of acquiring at higher spatial resolution a single subject (female, 22 years) was scanned using a 3D bFFE axial slab acquired in a transverse plane angled along the optic nerve at A; 2 mm isotropic resolution, 21 slices, compressed SENSE factor 4, TR/TE = 2.75/1.38 ms, FOV 128 × 192 × 42 mm^3^, dynamic scan duration 1.5 s, 220 dynamics, total scan duration 5 min 32 s; and also at B: 1.4 mm isotropic resolution (29 slices Compressed SENSE factor 6, TR/TE = 3.05/1.53 ms, FOV 128 × 186 × 42 mm^3^, dynamic scan duration 2.1 s, 160 dynamics, total scan duration 5 min 46 s using the same grey scale checkerboard presented for 0.5 s.

#### Somatosensory stimulus applied to the index fingers and big toe

Index finger stimulation funCI data were acquired from four healthy males (median age 30, range 28–43 years) and one female (24 years), who were the same participants as in the visual experiments already described.

The fingertip somatosensory stimulus consisted of periodic stimulation of the index fingertips of either the left or right hand where the stimulus was applied using a plastic fork to scrape the fingertip. The stimulus method was similar to the method we used previously to map the somatosensory cortex with fMRI [41], but the duration of stimulus was reduced from 6 s to 0.5 s. Participants lay supine inside the scanner bore with both hands palm upward and arms and hands propped with cushions and pads to minimize movements.

To stimulate different parts of the somatosensory cortex, we also scraped the pad of the right big toe in five participants. Data were acquired from five control participants (one female (24 years) and four males (age range 31–38 years). The participants bared their right foot, and lay supine inside the MRI bore with their knees supported by a wedge cushion. The participants were instructed to keep still and relaxed, close their eyes, and not to move their legs or feet. Five healthy participants were scanned using a dynamic bFFE sequence. During the bFFE sequence, the pad of the right big toe was scraped for 0.5 s with a plastic fork by a researcher to stimulate the somatosensory system. A visual prompt was displayed on an LCD screen in the scanner room out of sight of the participant to cue the researcher to perform the scraping task.

Acquisition parameters using bFFE for both finger and toe scraping were: a 3D non-selective balanced fast field echo sequence, TR/TE = 1.89/0.78 ms, 25° flip angle, compressed SENSE factor 4, 3 mm isotropic resolution, FOV = 240 × 240 × 189 mm^3^, sagittal acquisition, dynamic scan duration 1.89 s (220 dynamics and 5 dummy scans).

Acquisition parameters for the same experiment using fMRI were acquired using single-shot echo-planar imaging, SENSE = 2.9, multiband 3, TR/TE = 2000/30 ms, FOV 240 × 240 × 189 mm^3^, 3 mm isotropic resolution with 5 dummy scans and 220 dynamics. Stimulation was delivered to the left or right index finger as for the funCI experiment above.

#### Heat stimulus applied to the cheek

Data from an acquisition using heat stimulation applied to the cheek were collected from one healthy male control (23 years). Heat stimulation was applied to the right cheek via an MRI-compatible contact thermode (16 $\times$ 16 mm^2^, TSA-2 thermode, Medoc, Israel). MRI acquisition parameters for this experiment were a 3D non-selective bFFE sequence, TR/TE = 1.91/0.76 ms, 25° flip angle, compressed SENSE factor 4, 3.75 mm isotropic resolution, FOV = 240 × 240 × 187.5 mm^3^, sagittal acquisition, dynamic scan duration 1.2 s, duty cycle 16 scans (220 dynamics and 5 dummy scans). The thermode took 2.5 s to reach the target temperature (47 °C) and then was immediately switched off. This stimulus was reported as heat by the participant, but not as noxious heat.

#### Acquisitions for estimation of response functions

Data from funCI visual stimulation experiments used to calculate stimulus and contrast response functions were obtained from healthy participants, one male 29 years and one female 24 years old. This latter acquisition was repeated on a separate date, same time of day, in both of these participants in order to determine response function repeatability. For estimation of the funCI response function, which required higher temporal resolution, the same sequence was used as for visual stimulation, but acquired at 3.75 mm isotropic resolution, TR/TE 1.89/0.74 ms with 1.2 s dynamic scan time and total acquisition time of 4 min 35 s. The start of the stimulus was jittered from −0.3 s to 1.2 s with 0.1 s increments in semi-randomised order to reduce potential time-in-scanner or stimulus desensitization artefacts. Both phase and magnitude images were saved.

To determine how the response function altered with stimulus duration, data were acquired using 8 Hz flashing checkerboard using 3D bFFE (TR/TE = 1.90/0.74 ms, 3.75 mm iso, flip angle 25°, FOV = 240 × 240 × 187.5 mm^3^, dynamic scan duration 1.2 s, CS 8, 2 × NSA) with 0.45 contrast and stimulus durations of 0.1, 0.2, 0.3, 0.4 and 0.5 s. To determine the effect of changing contrast on the contrast response function, data were acquired using a 0.5 s duration stimulus with 8 Hz flashing checkerboard with greyscale contrasts of 0.25, 0.45 and 0.7.

The relationship between the peak amplitude of the conductivity response, determined by taking the second derivative of the fitted response function, and stimulus duration or contrast was calculated in Graphpad Prism 9.2.0 (GraphPad Software, Boston, MA) using simple linear regression.

### Image processing—conductivity reconstruction

Phase unwrapping: The measured transceive phase was unwrapped into the continuous phase as described previously [33], using Speedy rEgion-Growing Algorithm for Unwrapping Estimated Phase (SEGUE) [42]. SEGUE divides the $[\text{0,2}\pi )$ phase interval into six smaller intervals, determines connected 3D regions, and gradually enlarges the region with the largest border by unwrapping and merging the adjacent regions. SEGUE can provide similarly accurate results as the gold standard method, Phase Region Expanding Labeller for Unwrapping Discrete Estimates (PRELUDE) [43] and works 1.5 to 70 times faster than PRELUDE depending on echo time and anatomical regions [42], which makes SEGUE suitable for high-resolution phase unwrapping for MREPT.

Phase denoising: Denoising is often implemented for phase-based MREPT [44, 45] because the Laplacian operation is sensitive to noise [46]. As described previously [33], linear filters were found to either underestimate or overestimate the conductivity and permittivity values, while anisotropic diffusion filters performed better at the cost of computation time [45]. An adaptive nonlinear denoising filter was proposed to improve the image quality of reconstructed electrical properties at a reduced computation time [45], which uses parameters from a geometric model to differentiate noisy voxels from voxels on the edges in the three dimensions. This data-driven denoising filter is applicable to real and imaginary parts of the complex ${B}_{1}$ field, or its magnitude and phase, which will improve the conductivity and permittivity images to different degrees. In our experiment, the adaptive nonlinear filter was applied to the unwrapped phase, with number of iterations 400, constant integration 0.18, and Sigmoid diffusivity function.

**Fig. 1:**
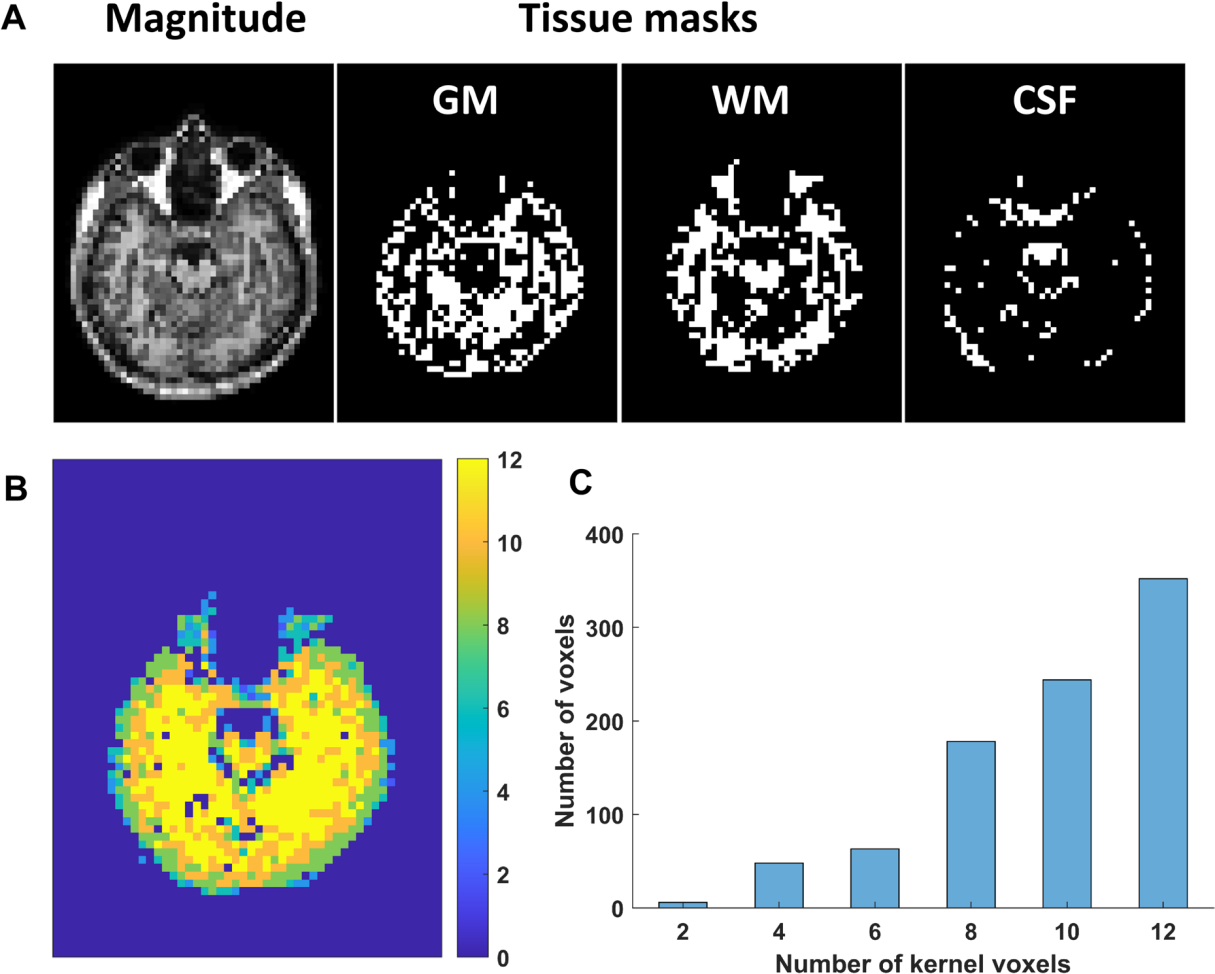
Example magnitude image and tissue masks and number of surrounding voxels for Laplacian estimation. **A** From left to right, magnitude image acquired using D T1-turbo field echo (TR/TE/TI = 5.89/2.76/850 ms, resolution 1 $\times$ 1 $\times$ 1 ${\text{mm}}^{3}$, sagittal slices, FOV 240 $\times$ 240 × 189 ${\text{mm}}^{3}$, flip angle 8$^\circ$ compressed SENSE factor 4) and coregistered to the bFFE magnitude image (resolution 3 × 3 × 3 mm^3^); Grey matter, white matter and CSF masks generated using the Brain Extraction Tool (BET) and FMRIB's Automated Segmentation Tool (FAST) segmentation from the FMRIB Software Library (FSL; FMRIB, Oxford, UK) [47]. **B** the number of included surrouding voxels when estimating the Laplacian of each voxel in Fig. 2. **B** shows a histogram of numbers of surrounding voxels in the kernels. ~ 87% voxels had ≥ six surrounding voxels for Laplacian estimation. ~ 95% of the active voxels in Fig. 2 had ≥ six surrounding voxels for Laplacian estimation

Brain tissue segmentation: Numerical calculation of the Laplacian involves a voxel ensemble/kernel around the target voxel. Voxels near tissue boundaries may have neighbouring voxels belonging to different tissue types. Here, T1W TFE scans were co-registered using SPM 12 (The Welcome Centre for Human Neuroimaging, UCL, London, UK), then segmented into three main tissue types, white matter (WM), gray matter (GM) and cerebrospinal fluid (CSF) using the Brain Extraction Tool (BET) and FMRIB’s Automated Segmentation Tool (FAST) segmentation from the FMRIB Software Library (FSL; FMRIB, Oxford, UK) [47] to help alleviate boundary artefacts in the conductivity calculation. An example of the masking used is shown in Fig. 1.

Numerical Laplacian: Calculation of phase-based MREPT conductivity was performed voxel-wise, as expressed below:

$$\sigma \approx \frac{\nabla^2 \varphi_\pm }{{2\mu_0 \omega }}$$

where $\sigma$ is conductivity, ${\varphi }_{\pm }$ denotes the transceive phase, ${\mu }_{0}$ is the magnetic permeability of free space, $\omega$ is angular frequency (Larmor frequency) and ${\nabla }^{2}$ is the Laplacian operator.

Within each tissue type, an average parabolic phase fitting method as described previously [33] was used to reduce artefacts [34]. Taking a row of phase voxels for a certain spatial dimension for example, parabolic fitting could be conducted with the target voxel and the voxels on its left or right side, leading to two quadratic expressions of phase around the target voxel. The second derivative of the target phase was assigned by the average of second derivatives of the two quadratic expressions. Similarly, second derivatives of the target phase in the other two dimensions can be calculated, to obtain the Laplacian. The kernel was limited to voxels which had close image amplitudes to the target voxel, to further reduce boundary artefacts. The kernel size had an effect on the noise in conductivity reconstruction [48] and the maximum kernel size of voxels was set as 9 × 9 × 6. For each target voxel, the fitted phase was only accepted if the correlation coefficient between fitted phase and measured phase was larger than 0.7 [34, 48]. The number of surrounding voxels included when estimating the Laplacian of each voxel is shown in Fig. 1B&C. After the Laplacian of the phase image was calculated, the conductivity image was obtained with angular frequency $\omega =2\pi \cdot 127.76\times {10}^{6}$ rad/s of the 3 T MRI scanner used in this study.

### Image processing—functional conductivity imaging

To generate the response function, ROIs were identified using the Desikan/Killiany atlas [49]. From within those ROIs voxel values where the conductivity was greater in the first ON condition than the OFF (baseline) condition were selected. The values of these voxels separately from grey and white matter were averaged at each time point across the time course. The conductivity response function was estimated (NumPy 1.21.0 [50]) via a 10th order discrete Laguerre polynomial fitted using the least squares method. For each stimulus cycle, the zero-time point was taken as the starting time of the stimulus. The conductivity value calculated from each dynamic scan was assumed to be at the halfway point of the scan acquisition (time to acquire half of k-space). By offsetting each stimulus cycle, the temporal resolution of the conductivity signal was improved up to 100 ms. In the case of the heat stimulus where the stimulus followed a longer time course than that used in the visual or finger/toe stimulation experiments, the response function was fitted to data from a time series where the stimulus onset was offset (jittered) from the start of each TR and the ROI of interest was the thalamus area.

The agreement of repeated measures of the response function (Fig. S2) was estimated in MedCalc for Windows, version 19.4 (MedCalc Software, Ostend, Belgium) using a single score one way intraclass correlation coefficient [51] using all repeat data from the two participants.

Conductivity images for activation mapping were processed in MATLAB R2018b (The MathWorks Inc, Natick, Massachusetts, USA) using a first level (general linear model) GLM and convolving the signal using the derived conductivity response function. The obtained T-value maps were co-registered to the high-resolution T1W images in SPM 12. Histogram smoothing was applied during estimation and reslice using 4th degree B-Spline interpolation. The co-registered T-values maps were overlaid on the T1W images for anatomical reference.

#### Image processing—fMRI

BOLD fMRI data were processed in SPM 12. The fMRI data were aligned and smoothed with 4 mm full-width half maximum before the first level general linear model using canonical haemodynamic response function. The activation T-value maps were co-registered and overlaid on the T1W images for anatomical reference following the same procedure as for conductivity activation maps.

## Results

### Functional conductivity imaging detects repeatable activation in the visual system

**Fig. 2:**
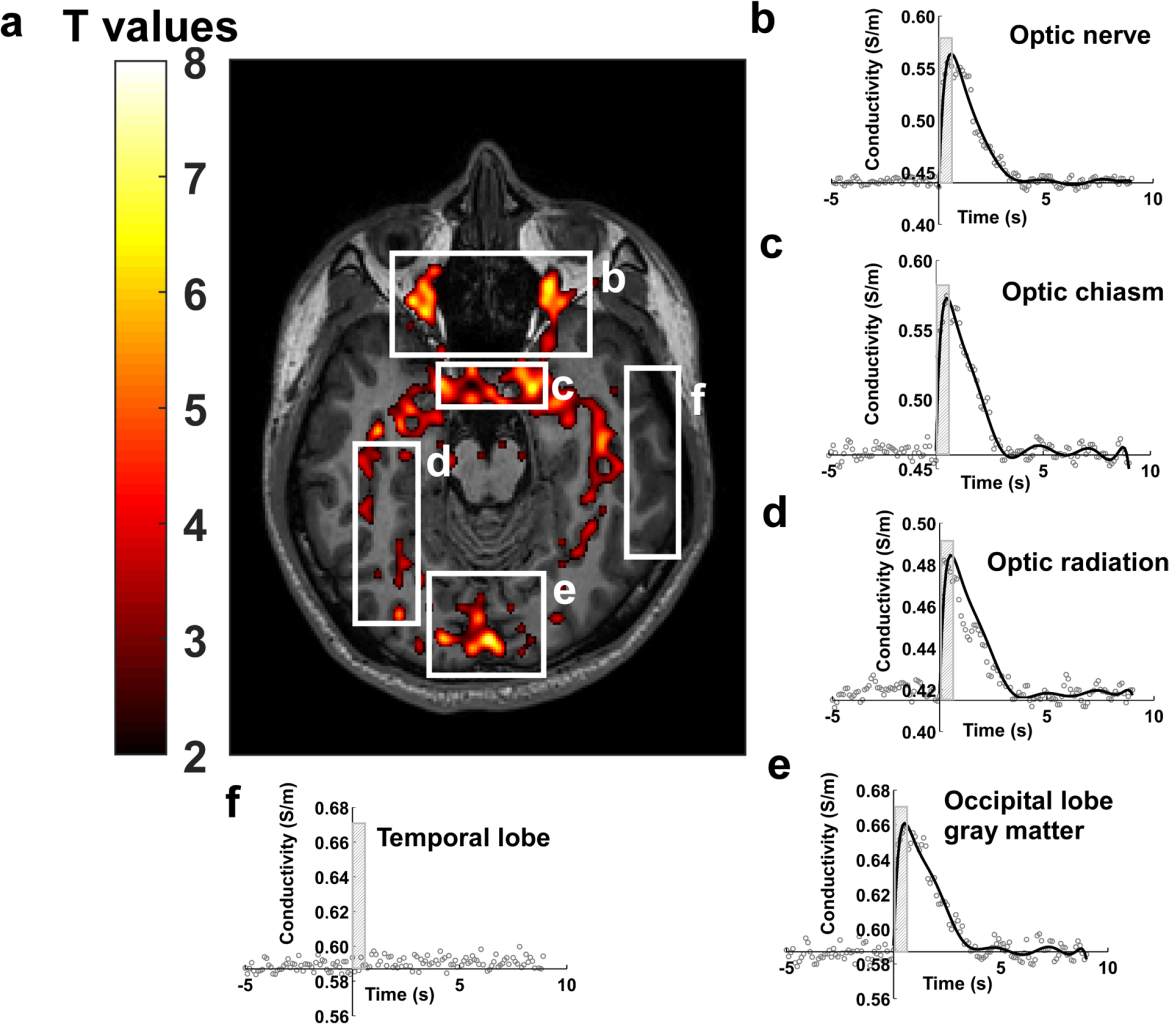
Visual stimulation produces measurable changes in tissue conductivity. **a** significant tissue conductivity changes in response to 0.5 s visual stimulation (8 Hz) using a grey scale (contrast = 0.45). Figure shows a single slice from the 3D acquisition selected on a plane to illustrate as much of the visual system as possible. **b**, **c**, **d**, **e** show changes in tissue conductivity estimated by fitting Laguerre polynomials using Numpy (1.21.0) in optic nerve, optic chiasm, optic radiation, occipital lobe grey matter from the image in a. Stimulus is shown as a hatched region. **f** null response from temporal lobe where tissue changes were not detected nor expected. Conductivity images were processed in MATLAB R2018b (The MathWorks Inc, Natick, Massachusetts, USA) using a first level (general linear model) GLM and convolving the signal using the derived conductivity response function. The obtained T-value maps were co-registered to the high-resolution T1W images in SPM 12 (The Welcome Centre for Human Neuroimaging, UCL, London, UK). Histogram smoothing was applied during estimation and reslice using 4th degree B-Spline interpolation. The co-registered T-values maps were overlayed on the T1W images for anatomical reference. Data were fitted in using Laguerre polynomials in NumPy (1.21.0) to obtain the conductivity response functions shown (see Methods for details). Activated voxels are those with T values > 2.2

**Fig. 3:**
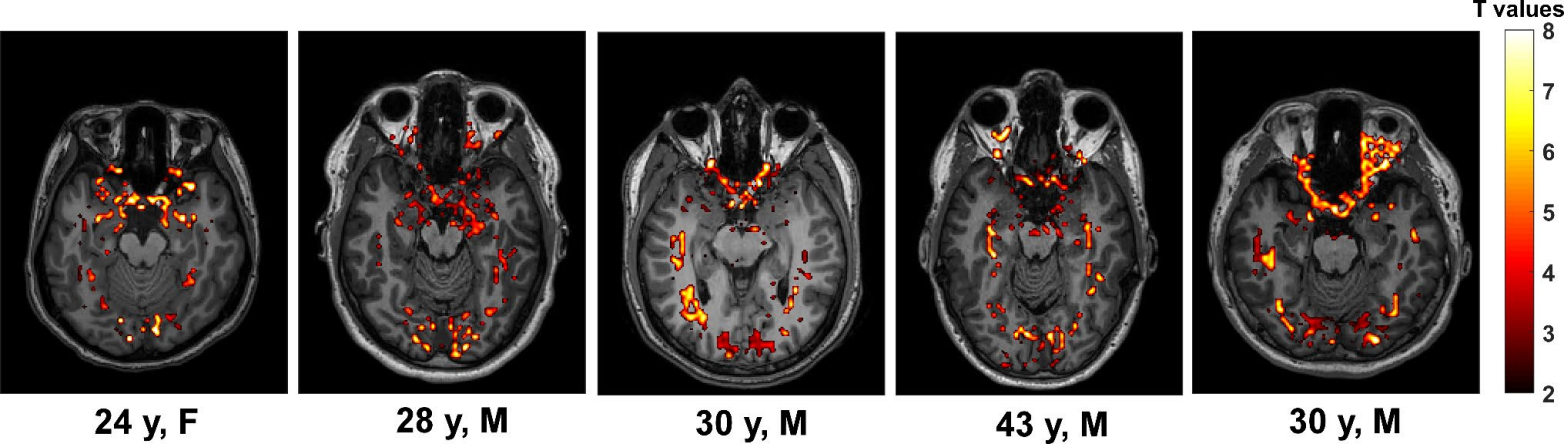
Visual stimulation shows consistent and robust tissue conductivity responses in five different individuals. Single axial slices from image pack showing significant changes in tissue conductivity in response to 0.5 s flashing greyscale checkerboard (contrast = 0.45). Conductivity images were processed in MATLAB R2018b (The MathWorks Inc, Natick, Massachusetts, USA) using a first level (general linear model) GLM and convolving the signal using the derived conductivity response function. The obtained T-value maps were co-registered to the high-resolution T1W images in SPM 12 (The Welcome Centre for Human Neuroimaging, UCL, London, UK). Histogram smoothing was applied during estimation and reslice using 4th degree B-Spline interpolation. The co-registered T-values maps were overlayed on the T1W images for anatomical reference

Stimulating the visual system with a flashing grey-scale checkerboard for 0.5 s produced significant increases (~ 0.1 S/m) in tissue conductivity in areas of the brain associated with the visual system, including around the optic nerve, optic chiasm, optic radiation and in the white and grey matter of the occipital cortex (Fig. 2). Individual responses to this stimulus are shown in native space in Fig. 3 where the experiment was repeated in five different individuals, indicating that the response to stimulus was similar in different participants. Additional slices from the participant shown in Fig. 2 can be viewed in Fig. S1.

The estimated funCI response function to this stimulus in the main regions of the brain involved in visual response is shown in Fig. 2. The time course of change in tissue conductivity showed an immediate and relatively large increase (0.1 S/m above baseline) in tissue conductivity that reached peak amplitude shortly after the end of the stimulus and decayed rapidly, returning to baseline levels within ~ 3–4 s of stimulus cessation (Fig. 2b–e). By contrast, there was no significant response to this stimulus in a region of the brain not associated with visual response, such as temporal lobe (Fig. 2f). The relative change in signal depended on the baseline tissue properties with increases in white matter in the order of ~ 20% and grey matter ~ 17%. These responses were measured at the same time of day on two different days in two different participants as well as twice on each of these days in one of the participants (Fig. S2) and found to be robustly repeatable with intraclass correlation coefficients equal to or greater than 0.89 (Fig. S2) signifying good to excellent repeatability. Information on phase stability can be found in supplementary information (Fig. S3).

**Fig. 4:**
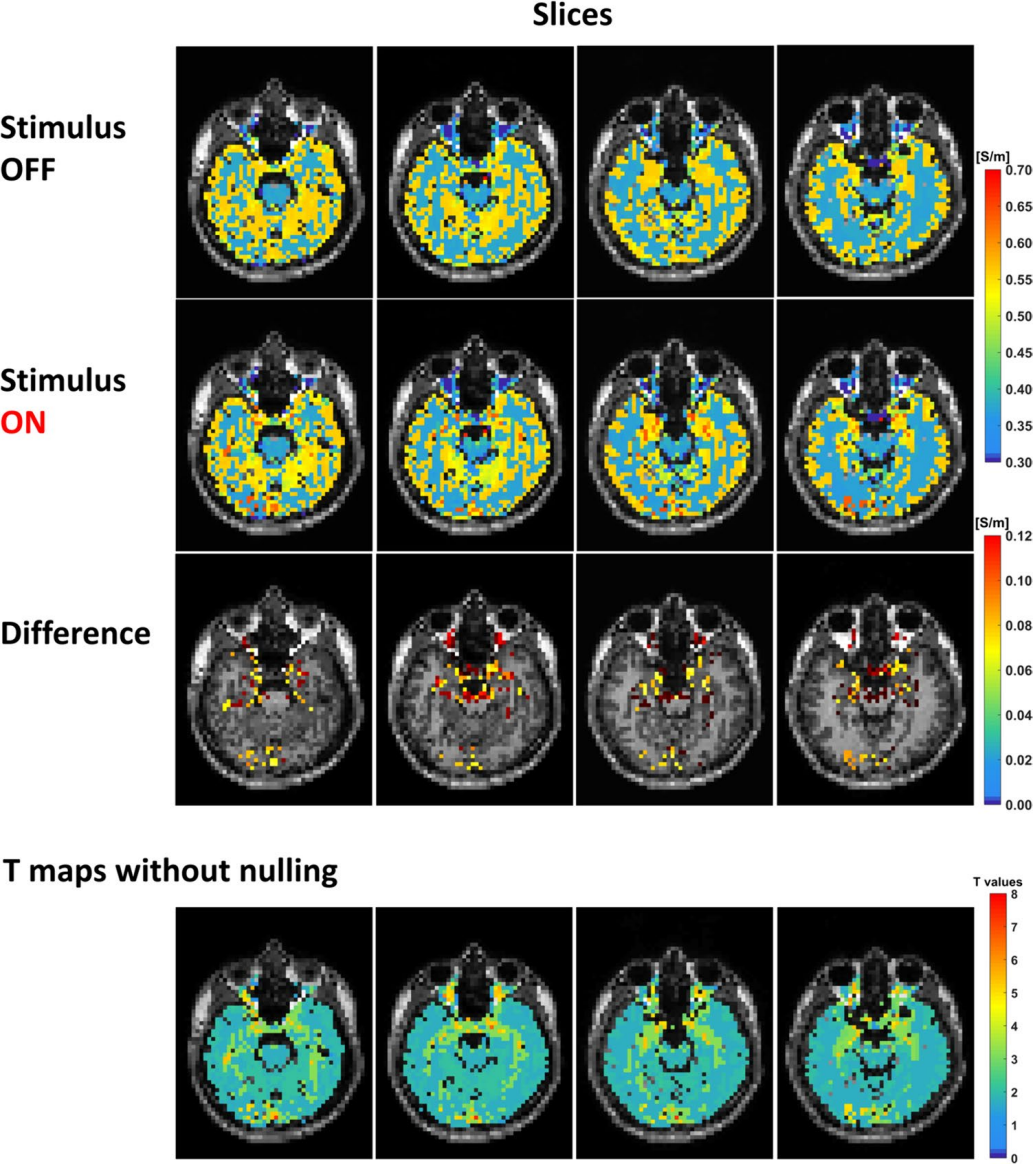
Axial slices showing conductivity maps of 0.5 s visual stimulus with stimulus OFF and ON, the difference between them and the conductivity t-map without nulling. Axial slices from image pack showing significant changes in tissue conductivity in response to 0.5 s flashing greyscale checkerboard (contrast = 0.45). Conductivity images were processed in MATLAB R2018b (The MathWorks Inc, Natick, Massachusetts, USA). Conductivity maps of all the OFF dynamic scans and ON dynamic scans were averaged separately in native space. The difference map was obtained by subtracting the averaged conductivity map of OFF sessions from the averaged conductivity map of ON sessions, then overlayed on the T1w map in native space

The data from one participant are displayed as conductivity maps (Fig. 4) in the condition where the stimulus was OFF (top row), where it was ON (second row), with the difference between these two maps shown below (third row). T-maps without nulling of voxels that did not reach significance are shown at the bottom.

**Fig. 5:**
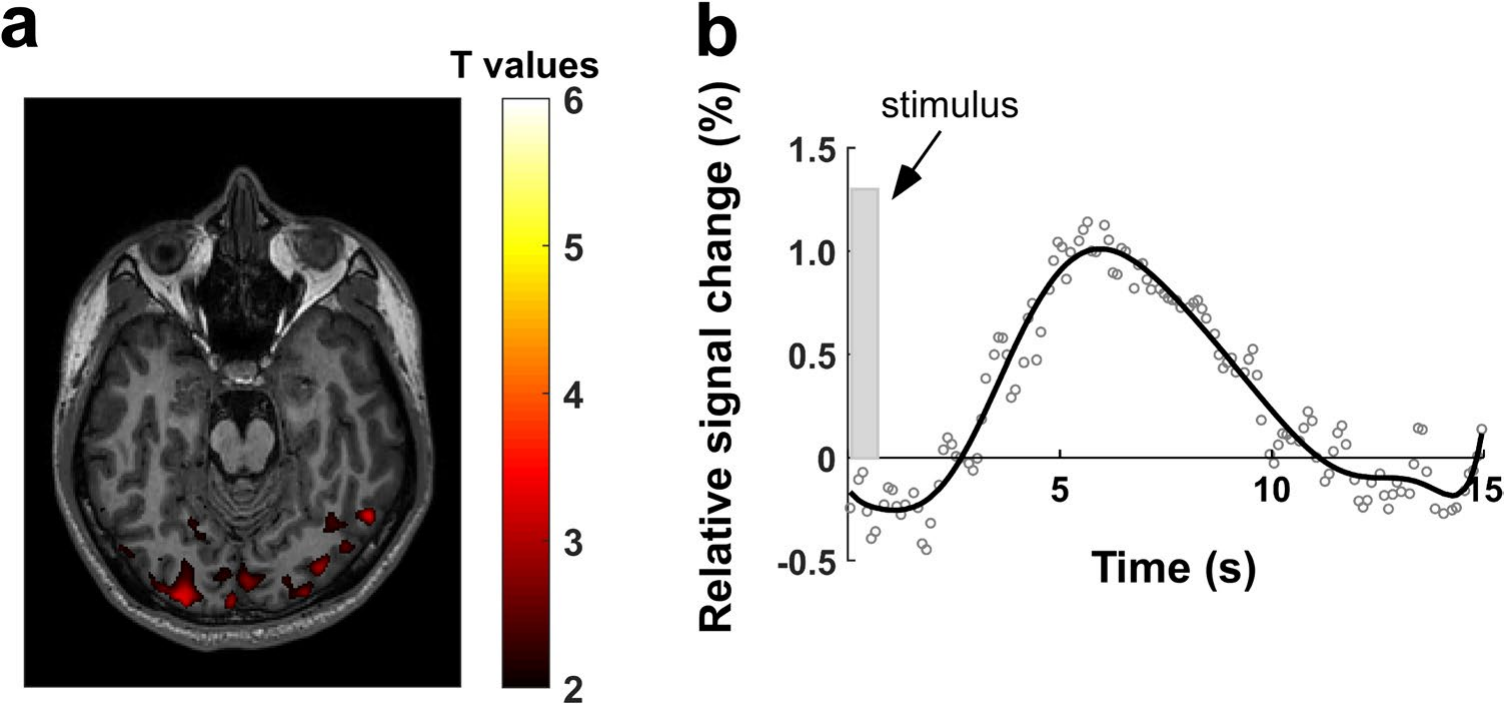
BOLD response to 0.5 s greyscale checkerboard visual stimulation. **a** Axial slice showing functional (BOLD) activation in response to 0.5 s visual stimulation (8 Hz) using a grey scale (contrast = 0.45). Activation T-value maps were co-registered and overlaid on the T1W images for anatomical reference. Adjustment for multiple comparisons was not used in this case as no voxel survived that treatment. **b** Timecourse of change of signal in response to stimulus and fitted response function

The activation map produced using funCI was distinct from that produced with an identical stimulus and total acquisition time but acquired using conventional BOLD echo-planar imaging (Fig. 5A). Specifically, in addition to the expected occipital lobe response that can be visualised with the BOLD technique, funCI was also able to detect activation in other parts of the visual system. The relative change of the BOLD response to the 0.5 s stimulus showed the expected small increase in signal of a little over 1% above baseline. The response function (Fig. 5B) followed the expected BOLD response [52].

### The impact of higher acquisition resolution

**Fig. 6:**
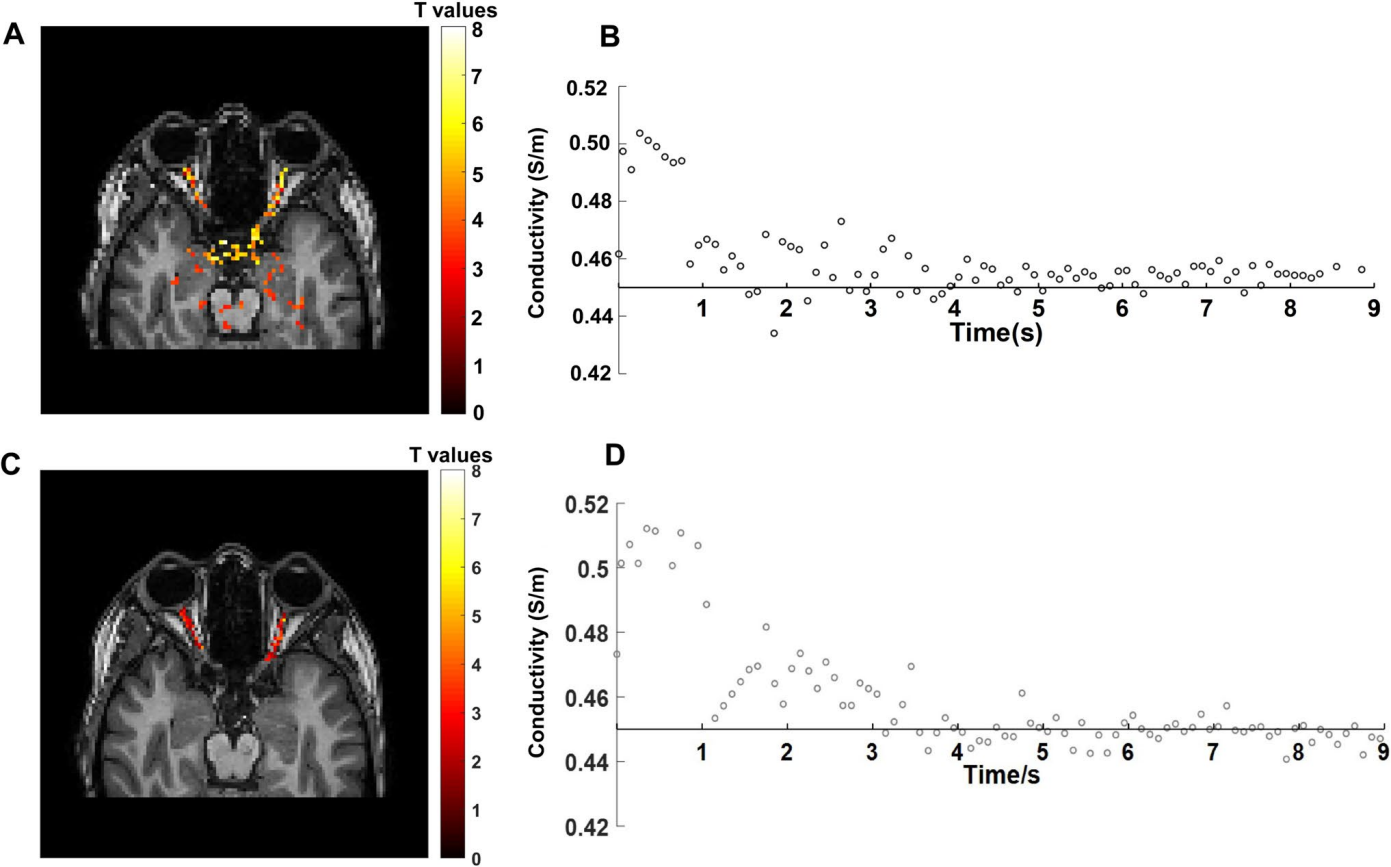
funCI of the optic nerve. **A** Conductivity map from a single axial slice angled along the optic nerve acquired at 2 mm isotropic resolution shown without masking. **B** Timecourse of conductivity changes in the optic nerve from data acquired at 2 mm isotropic. **C** Conductivity map from a single slice angled along the optic nerve acquired at 1.4 mm isotropic resolution, masked to show only optic nerve. **D** Timecourse of conductivity changes in the optic nerve from data acquired at 1.4 mm isotropic (area shown in red). Acquisition parameters for A were 2 mm isotropic, CS 4, TR/TE = 2.75/1.38 ms, dynamic scan duration 1.5 s, 220 dynamics, total scan duration 5 min 32 and for C; 1.4 mm isotropic, CS6, TR/TE = 3.05/1.53 ms, dynamic scan duration 2.1 s, 160 dynamics, total scan duration 5 min 46. The response functions were generated from voxels in the optic nerve where values from the first stimulus ON conditions were greater than in the stimulus OFF (baseline) condition. Conductivity values were averaged for each time point across the time course, and the data fitted using Laguerre polynomials in NumPy (1.21.0) to obtain the conductivity response functions shown

Data acquired using a higher resolution image pack focussed on the optic nerve region is shown in Fig. 6 with the associated tissue masks shown in Fig S4. The top panel shows the areas of significant change in conductivity in images acquired at 2 mm isotropic resolution. Activated voxels are those with T values > 2.2. The corresponding timecourse of conductivity response is shown top right. The bottom left panel shows data acquired with 1.4 mm isotropic, masked to show only significant (T > 2.2) activation in the optic nerve, with the corresponding time course of conductivity response shown bottom right.

### Activation response amplitude scales with stimulus duration and contrast level

**Fig. 7:**
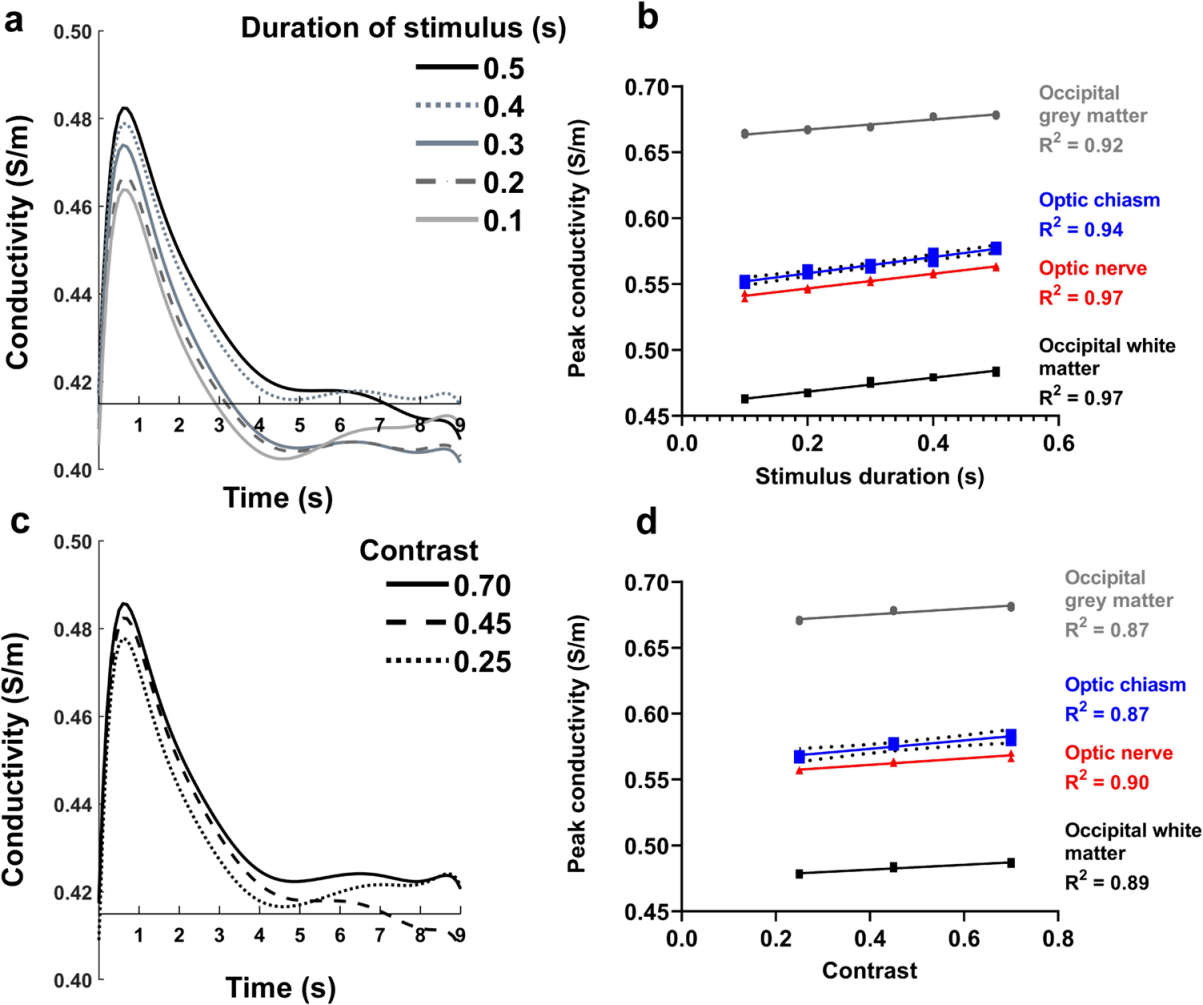
Increasing stimulus duration or contrast increases peak tissue conductivity. **a** exemplar response functions in occipital lobe white matter from one individual with increasing stimulus duration from 100 to 500 ms in 100 ms increments. **b** peak tissue conductivity levels from two individuals in response to increasing stimulus duration in four areas of the visual system fitted with simple linear regression. **c** exemplar contrast response functions in occipital white matter from one individual with increasing contrast levels. **d** peak tissue conductivity levels from two individuals in response to increasing stimulus contrast in four areas of the visual system fitted with simple linear regression (GraphPad Prism 9.2.0). Responses are shown from the first acquisition at stimulus onset. Response functions were fitted using Laguerre polynomials in NumPy (1.21.0) to obtain the conductivity response functions shown

We then tested to see if the response to the stimulus would scale with the stimulus duration and/or with visual contrast similar to the seminal experiment undertaken by Boynton et al*.* investigating the BOLD response [52]. Accordingly, the responses to stimulus durations varying from 0.1 s to 0.5 s in 0.1 s increments, and three different levels of greyscale contrast were measured in different areas of the brain in two participants. Figure 7 shows the stimulus and contrast responses from optic nerve and occipital lobe white and grey matter to increasing stimulus duration or increasing contrast. The response function was characterised on 100 ms time resolution increasing rapidly immediately at stimulus onset, peaking 100–200 ms after cessation of the stimulus and returning to baseline around 3 s after peaking, although the time to return to baseline was longer with the longer or stronger stimuli (Fig. 7A&C). The simple regression relationship was linear across the range measured, with the coefficients of variation (R^2^) indicating that the change in stimulus duration or contrast accounted for the majority of the variance in the peak signal intensity (Fig. 7B&D).

This investigation also revealed that funCI is sensitive to relatively short stimuli, with consistent changes in conductivity able to be detected with only 100 ms of visual stimulus (Fig. 7A).

### Somatosensory stimulation consistently elicits somatosensory circuit activation maps

**Fig. 8:**
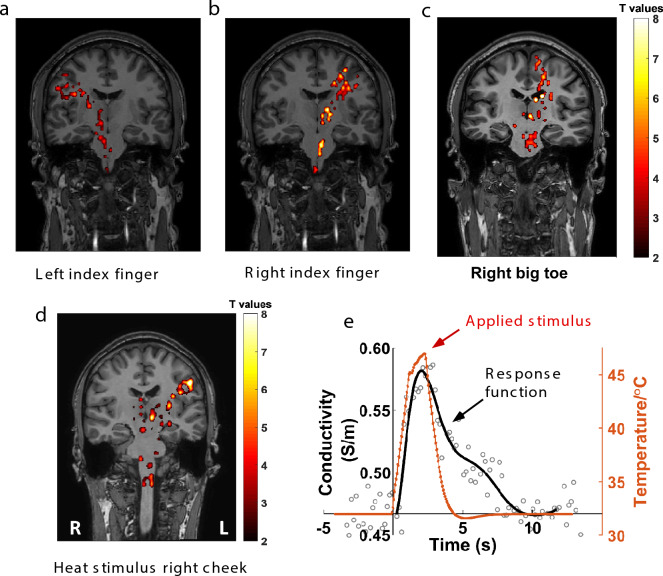
Sensory stimulation detected using functional conductivity imaging. **a** Coronal slices from image pack showing significant changes in tissue conductivity in response to scraping the left and **b** right index finger for 0.5 s. **c** Single coronal slice from image pack showing significant changes in tissue conductivity in response to scraping the pad of the right big toe. **d** Single coronal slice from image pack showing significant changes in tissue conductivity in response to a thermode generated heat stimulus applied to the right cheek ramped up over 2.5 s as shown in. **e** Timecourse of thermode temperature change (red) shown with conductivity response (grey circles) and the response function fitted using Laguerre polynomials (black) with Numpy (1.21.0). Activated voxels are those with T values > 2.2

**Fig. 9:**
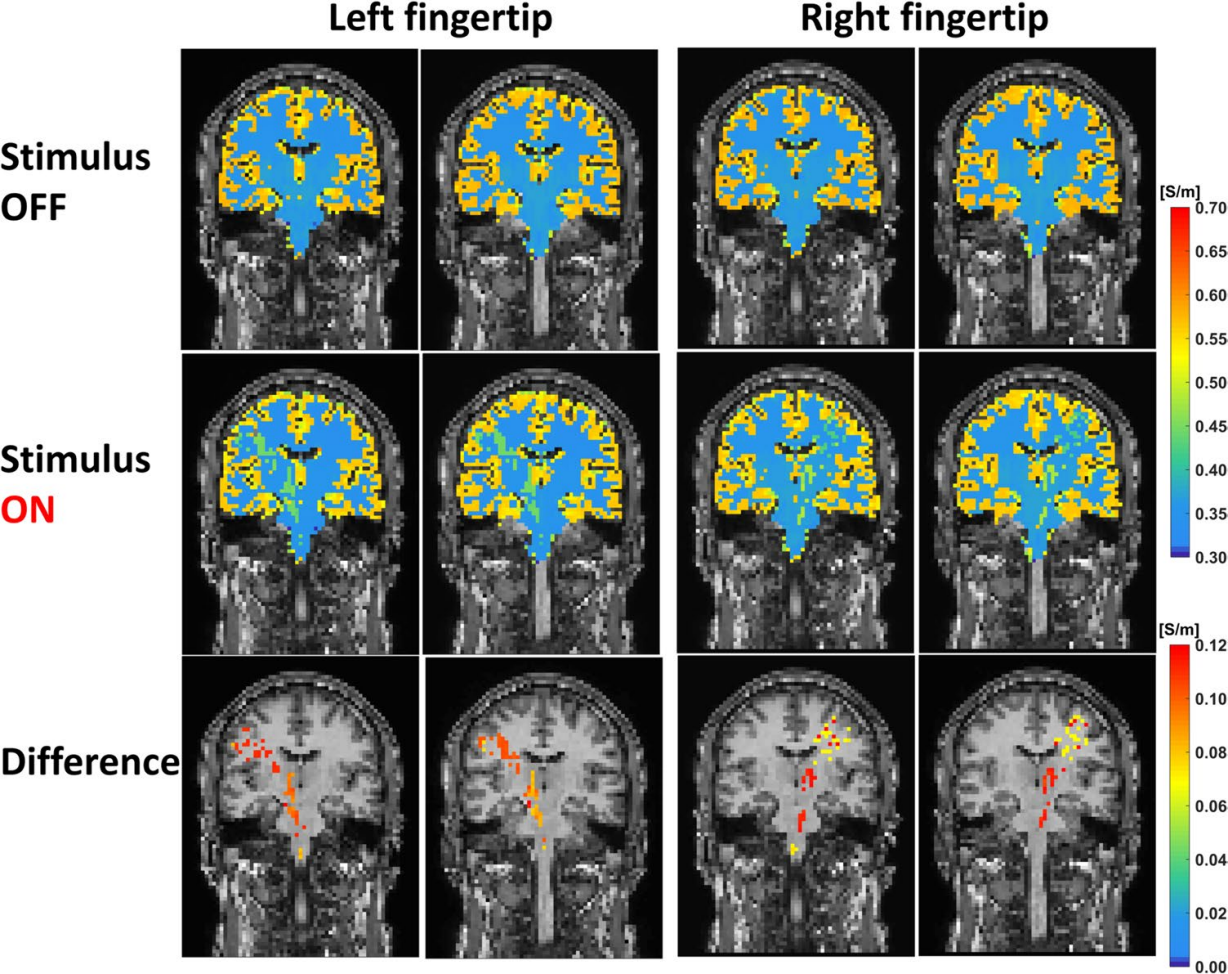
Coronal slices of sensory stimulation maps showing conductivity values with stimulus OFF and ON and the difference map. Coronal slices from image pack showing conductivity maps in response to scraping the left and right index fingers for 0.5 s. Top row, stimulus OFF; middle row, stimulus ON; bottom row, conductivity difference map. Conductivity maps of all the OFF dynamic scans and ON dynamic scans were averaged separately in native space. The difference map was obtained by subtracting the averaged conductivity map of OFF sessions from the averaged conductivity map of ON sessions, then overlayed on the T1w map in native space

To determine the ability of funCI to detect activation in other neural systems, we applied to five participants the same MR acquisition protocol where either their left or right index finger was scraped with a plastic fork to stimulate the somatosensory system. This produced similar activation patterns across the somatosensory network (Fig. 8A and B) with activation including corticospinal tracts, thalamus and the area of the right or left somatosensory cortex mapping to the finger being scraped (also shown as ON vs OFF and difference conditions in a single participant in Fig. 9). Response maps to finger scraping are shown in native space for five participants in Fig. S5, indicating that the response is robust and repeatable. For comparison, the BOLD response to the same stimulus is shown in Fig. S6.

Scraping the right big toe with a plastic fork in five different participants produced activation in the area of the somatosensory cortex corresponding to the right big toe homunculus (Fig. 8C).

Applying a longer ramped up/ramped down heat stimulus to the right cheek produced a response in the area of the left somatosensory cortex mapping to the face as well as the expected activation in the thalamus and corticospinal tracts (Fig. 8D). Due to the longer stimulus time (Fig. 8E) a different response function using 10th-order discrete Laguerre polynomials was fitted to the data averaged across the thalamus area of the slice as shown in Fig. 8D&E.

## Discussion

Here, through activation induced responses to visual and somatosensory stimuli (Figs. 2 and 8), we have shown that tissue conductivity changes measured with functional conductivity imaging can be used to map functional activation pathways in the brain. The advantages of using funCI include that it is quantitative, delivering repeatable measurements with conductivity response function peak amplitudes that scale with the duration of the stimulus (Fig. 7). It delivers consistent activation, showing near-identical response functions to visual stimulation in the same person on different days (Fig. S2). The method detects activation in both grey and white matter, making it a method that can be used to map entire functional pathways in the brain and it is sensitive to small stimuli, responding well to only 100 ms of visual stimulus (Fig. 7). The dynamic repetition time is similar to that of multiband EPI (1.2 s in this case) and the whole brain spatial resolution (3 mm isotropic) is also similar to that used in many fMRI acquisitions. Therefore, funCI offers a useful alternative to BOLD fMRI for the spatial and temporal mapping of brain activity.

FunCI is a sensitive method, detecting gray-scale visual stimulation durations as short as 100 ms (Fig. 7). This compares favourably with BOLD fMRI where a similarly weak 0.5 s stimulus is barely detectable (Fig. 2 vs Fig. 5 and Fig. 8 vs Fig. S6). A full visual field stimulus with a contrast of 0.63 presented for 0.5 s has been reported previously to elicit a BOLD response of around 0.75% [53]. The stimulus presented here (Figs. 2, 3 & 6) was even weaker at a contrast of 0.45 and could barely be detected using BOLD EPI (Fig. 5). By contrast, the response using funCI was robust when similarly presented for 0.5 s and easily detected when presented for as little as 100 ms (Figs. 2 and 7).

As the stimulus duration increase it was possible to observe the appearance of a “tail” on the response timecourse (Fig. 7). This can be seen particularly well in the response to the heat stimulus applied to the face (Fig. 8) where a 2.5 s stimulus was applied due to the time taken to ramp up the thermode to the desired temperature. This may represent a different, additional biophysical response thatmay represent a drawback of funCI if it is applied to longer and more complex stimuli. However, the fast response to short stimuli does auger well for the use of funCI in detecting subtle brain activation arising from such relatively weak stimuli.

Other researchers have reported detecting changes in the EPT signal on stimulation including motor stimulation (fist clenching [35]; finger tapping [54] and visual stimulation [36, 55], including changes of similar magnitude (0.1 S/m). These observations were either made on single slice acquisitions, limiting the ability to visualise entire circuitry, or using relatively long stimulation times which limit the ability to detect fast changes in conductivity.

The response from white matter is of particular novelty. For example, the optic nerve area, optic chiasm and optic radiation are all significantly activated by visual stimulation (Figs. 2, S2 and 3) and the corticospinal and thalamic relays of the somatosensory system can clearly be visualised following sensory stimulation of the index finger, big toe or cheek (Fig. 8). The signal is confined to the activated pathways, with non-activated areas of the brain, such as the temporal lobe following visual stimulation, showing no significant change in tissue conductivity (Fig. 2f and Fig. S2). In the white matter, the relative signal increases are large, given the lower baseline (resting) tissue conductivity of white matter cf. grey (0.42 ± 0.02 S/m cf. 0.64 ± 0.03 S/m; [33]) meaning that activation in white matter pathways feeding activated grey matter can also easily be visualised with funCI.

The whole brain scan used here was acquired at 3 mm isotropic resolution. Coupled with the Laplacian estimation this resulted in some smoothing (e.g., around the optic nerve area in Fig. 2). However, acquiring funCI scans at higher resolution (e.g., 1.4–2 mm isotropic, Fig. 6) demonstrated that funCI has sufficient spatial resolution to detect conductivity changes in small structure such as the optic nerve, albeit with limited anatomical coverage.

The acquisitions at different spatial resolution also illustrate the effect of partial voluming, where the larger voxels used here for whole brain coverage are likely to contain more than one tissue type. The higher resolution scans (Fig. 6, Fig. S4) are less likely to invoke partial volume effects and are more suitable for measuring small tissues like the optic nerve, but essentially have decreased signal to noise (the voxel volume is ten-fold smaller), making the phase estimation possibly less reliable than in the larger voxels (although the 3 mm^3^ acquisition is signal rich). The time course of response measured in the higher resolution images is similar to that from the 3 mm^3^ acquisition suggesting that the conductivity change is reasonably robust to voxel size, although there may be added uncertainty in the quantitation of the change. The absolute measurement may be less reliable in areas of small grey matter volume, such as the optic nerve, due to partial voluming.

The temporal response to the stimuli is on a different time scale to that of the traditional BOLD response [56]. Comparing the time course of the response functions measured using funCI with those measured using BOLD (Fig. 4) shows the funCI response (Figs. 2, 2S and 4) peaking much earlier relative to the cessation of the stimulus and returning to baseline by the time the BOLD response is peaking. BOLD peaks around 5–6 s after 8 Hz stimulation [57, 58]. Earlier response of tissue conductivity in the occipital cortex on visual stimulation as compared to BOLD has previously been reported [36] although the response function was not measured in that case. Blood flow changes in response to stimulus are known to occur on a timescale that is highly reproducible across individuals and, although it does vary with brain region [59], the response time of blood flow to activation occurs on a seconds time scale, peaking much later than the response seen here with funCI. Further, BOLD effects on electrical conductivity have been reported to result in decreases in electrical conductivity rather than increases as oxygenated blood (being diamagnetic) is less conductive than deoxygenated (paramagnetic) blood [38]. The electrical conductivity of blood has been measured as 1.25 S/m [60] which is somewhat higher than grey matter but blood volume makes up only around 3–4% of grey matter at rest and increases by no more than 50% on vigorous visual stimulation [61]. It would therefore seem that the “initial dip” seen in some fMRI experiments, which is due to increased deoxy vs oxy haemoglobin levels is unlikely to be able to account for a 0.1 S/m increase in conductivity as deoxyhemoglobin levels only increase (if at all) by around 6% [62]. This means that the conductivity of deoxygenated blood would need to be around four and a half times greater than the conductivity of oxygenated blood in order to increase conductivity by 0.1 S/m solely due to this effect; this is unrealistic given the reported values [63]. Of course this could be tested via an experiment such as that undertaken to test the vascular contribution to the diffusion fMRI signal [64].

Neural electrical activity generally occurs within milliseconds of the stimulus with EEG recorded visually evoked potentials typically producing responses in the range of 75–300 ms post-stimulus [65]. Electrical activity is also associated with the movement of ions and changes in free water, all of which could potentially contribute to changes in tissue electrical conductivity and to the signal detected here.

What could cause the underlying signal change seen here? Petridou et al. showed, in an organotypical slice preparation from prenatal rat brain with no blood flow, that neural activity alone, was sufficient to induce phase changes in the magnetic resonance signal [10]. There have been several investigations and theoretical studies made on whether the phase changes induced by electrical activity can be realistically detected (above noise) in MRI or not. Phantom work has shown that there is a linear relationship between the phase change measured in a spin echo experiment and electrical conductivity [36]. In rat tissue cultures a phase change due to the electrical signal of 0.2–0.8° was reported [10] while models in human neurons have reported phase changes up to 0.32°, depending on the type of neuron being studied [66]. The phase change corresponding to 0.1 S/m, as measured here, is of the order of 0.8°. This compares with the phantom phase noise in our study, calculated according to Scott et al. [67] of 0.271° indicating that here, the phase change is detectable above the intrinsic phase noise. The phase “noise” in vivo is higher due to natural variations in the signal, similar to those seen in resting-state BOLD (Fig. S3). Whether the measured response is due completely to neural activity remains to be explored.

The similar phase changes seen in grey and white matter of 0.1 S/m argue against the influence of blood flow effects on the phase. Blood flow changes are known to be significantly greater in grey than white matter [3] so we would expect to see that play out in the data were it to be the case. Similarly, cardioballistic artefacts or respiratory movement might be expected to contaminate areas of the brain primarily around large arteries, or the whole brain, and result in a spatial pattern different to that seen here, where response to a visual task reflects the visual system; this is also reflected in the spatial pattern seen with somatosensory stimulation.

Neural activation is known to be associated with cell swelling [68] where shrinkage of the extracellular space has been reported to affect the MRI signal, for example by altering the diffusion of free water [69] or through altering signal intensity [70, 71] but in both these cases the timecourse of signal change is much slower than that seen here with funCI. Further work is needed to fully understand the biophysical basis of the signal changes reported here.

## Conclusion

In summary, funCI is a quantitative, sensitive and repeatable application for non-invasive, detection of brain activity with temporal and spatial acquisition resolution comparable to traditional fMRI. Importantly, it allows quantitative measurement of the full brain circuitry responding to a functional task, allowing more wholistic understanding of brain and behaviour, including in the study of brain connectivity [72]. Application of funCI should result in a new era of discovery of functional brain circuitry activation.

## Supplementary Information

Below is the link to the electronic supplementary material.

Supplementary file1 (TIF 2269 KB)

Supplementary file2 (TIF 786 KB)

Supplementary file3 (TIF 612 KB)

Supplementary file4 (TIF 203 KB)

Supplementary file5 (TIF 2674 KB)

Supplementary file6 (JPG 994 KB)

Supplementary file7 (DOCX 16 KB)

## Data Availability

Imaging data generated during this study is available from the University of New South Wales data repository http://handle.unsw.edu.au/1959.4/resource/collection/resdatac_1259/1, 10.26190/M3F9-1N36.

## Code availability

Matlab code and further information on processing pipelines is available from the authors upon reasonable request.
