# Supplementary figures and images for: Functional conductivity imaging: quantitative mapping of brain activity
Source: Phys Eng Sci Med. 2024 Sep 11;47(4):1723–38. doi: 10.1007/s13246-024-01484-z (PMC11666624; doi:10.1007/s13246-024-01484-z)

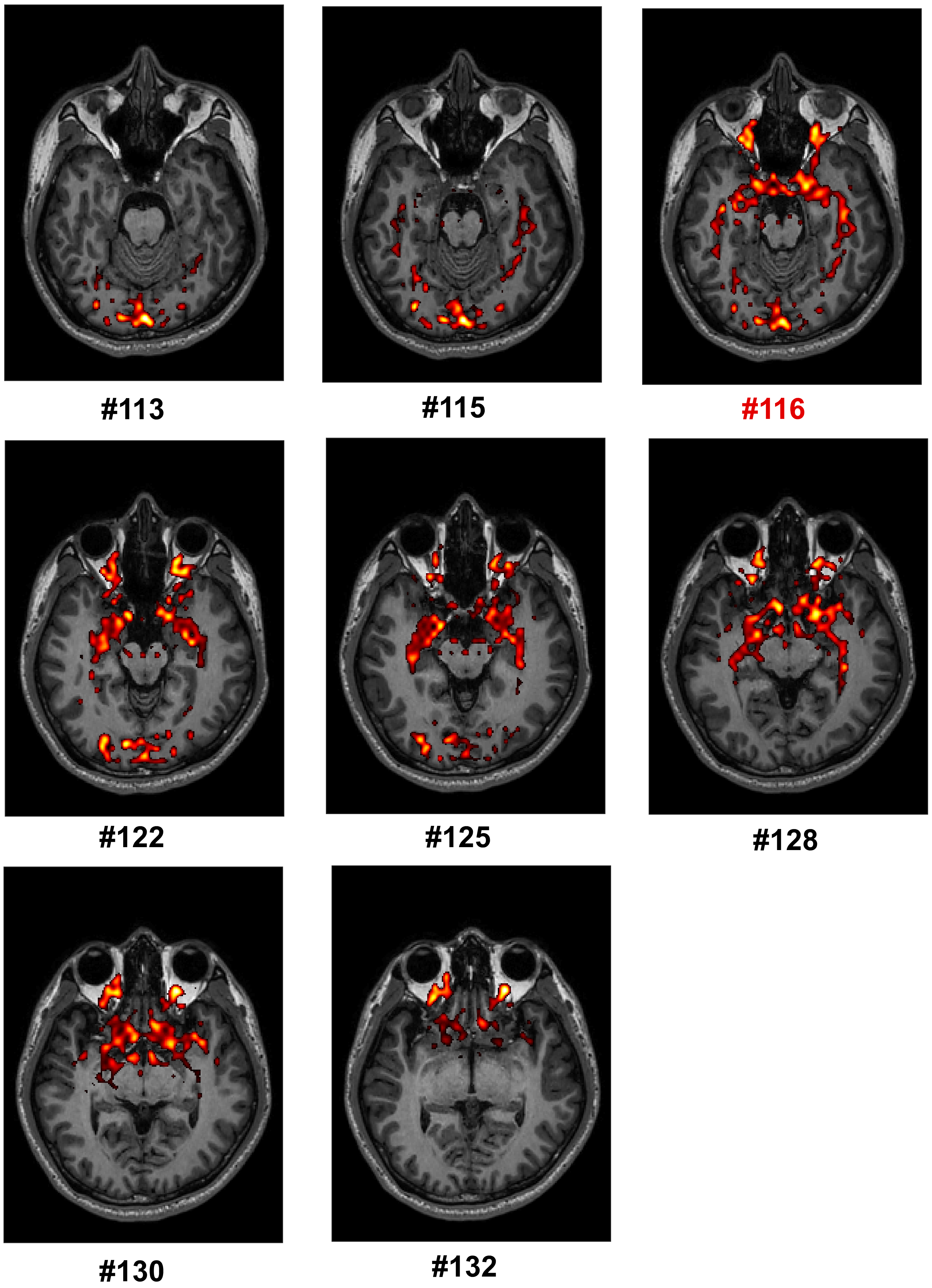

Supplement: Supplementary file 1 — Supplementary file1 (TIF 2269 KB) [file 13246_2024_1484_MOESM1_ESM.tif]

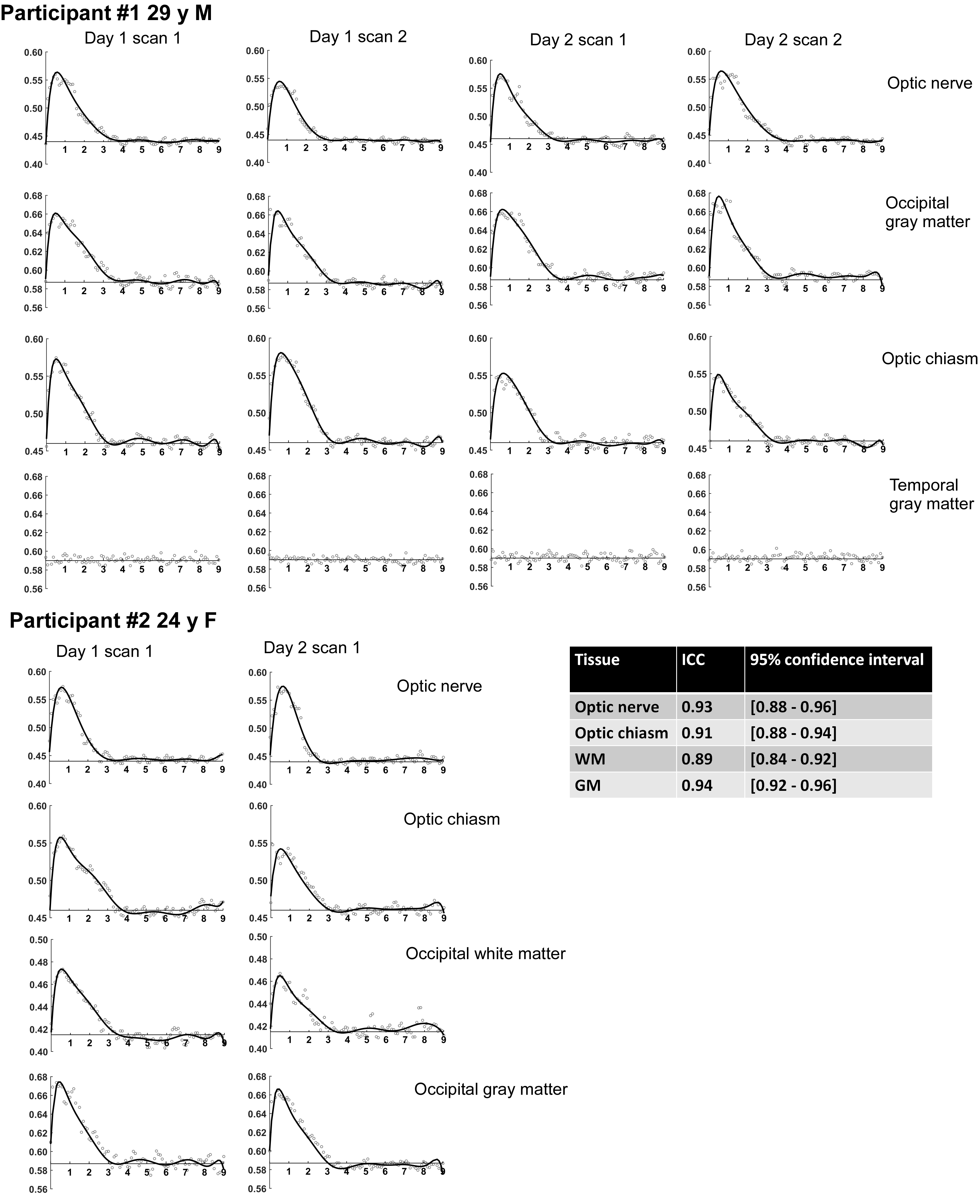

Supplement: Supplementary file 2 — Supplementary file2 (TIF 786 KB) [file 13246_2024_1484_MOESM2_ESM.tif]

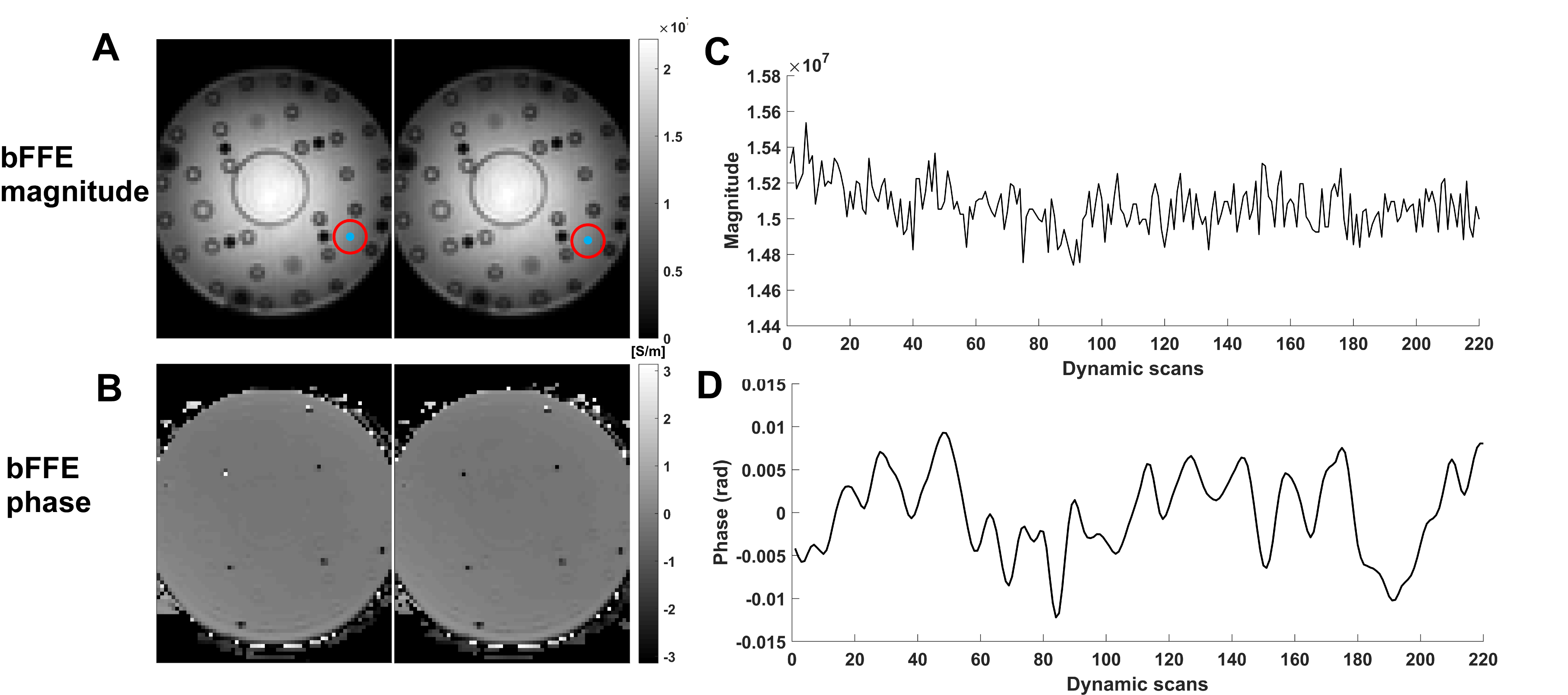

Supplement: Supplementary file 3 — Supplementary file3 (TIF 612 KB) [file 13246_2024_1484_MOESM3_ESM.tif]

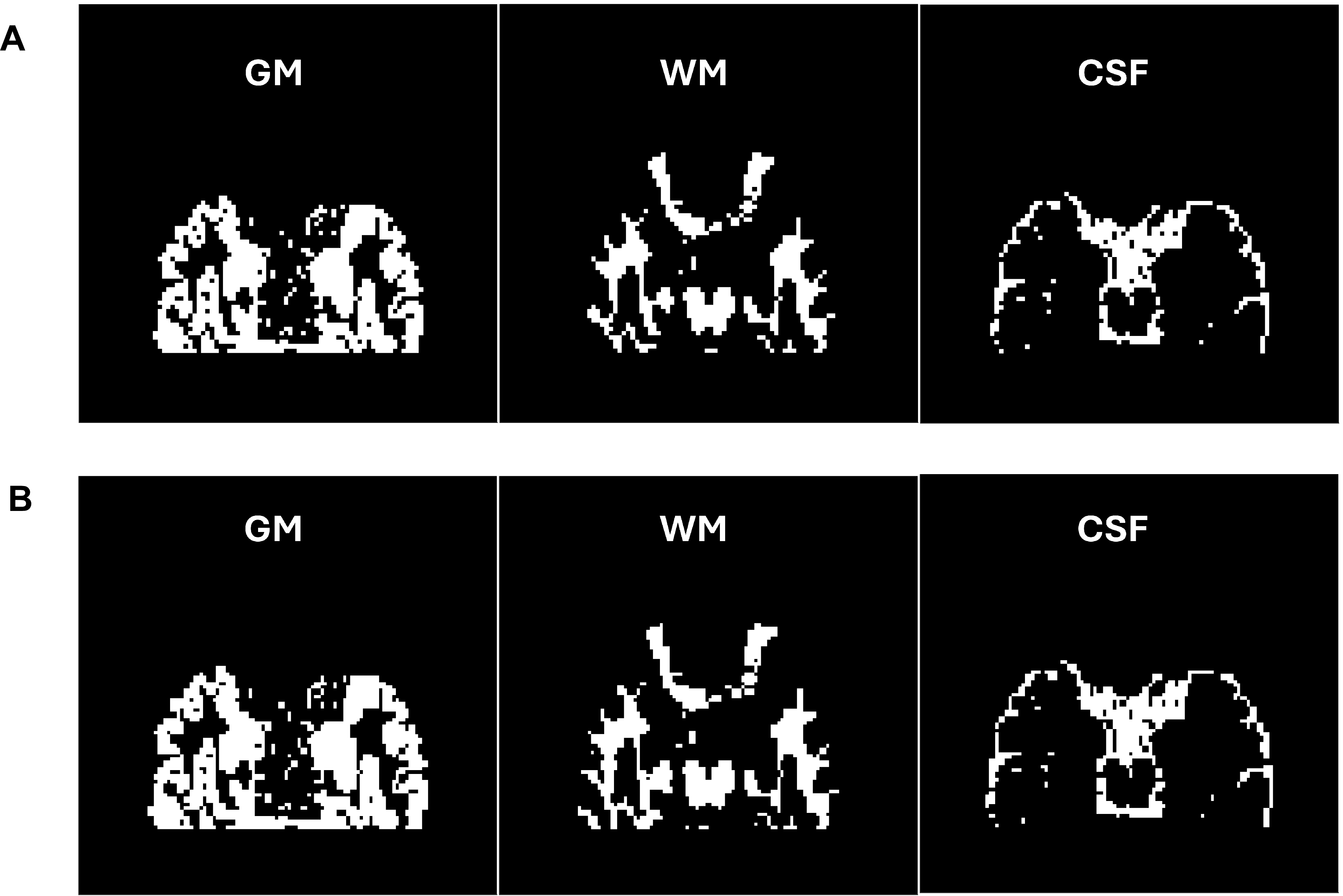

Supplement: Supplementary file 4 — Supplementary file4 (TIF 203 KB) [file 13246_2024_1484_MOESM4_ESM.tif]

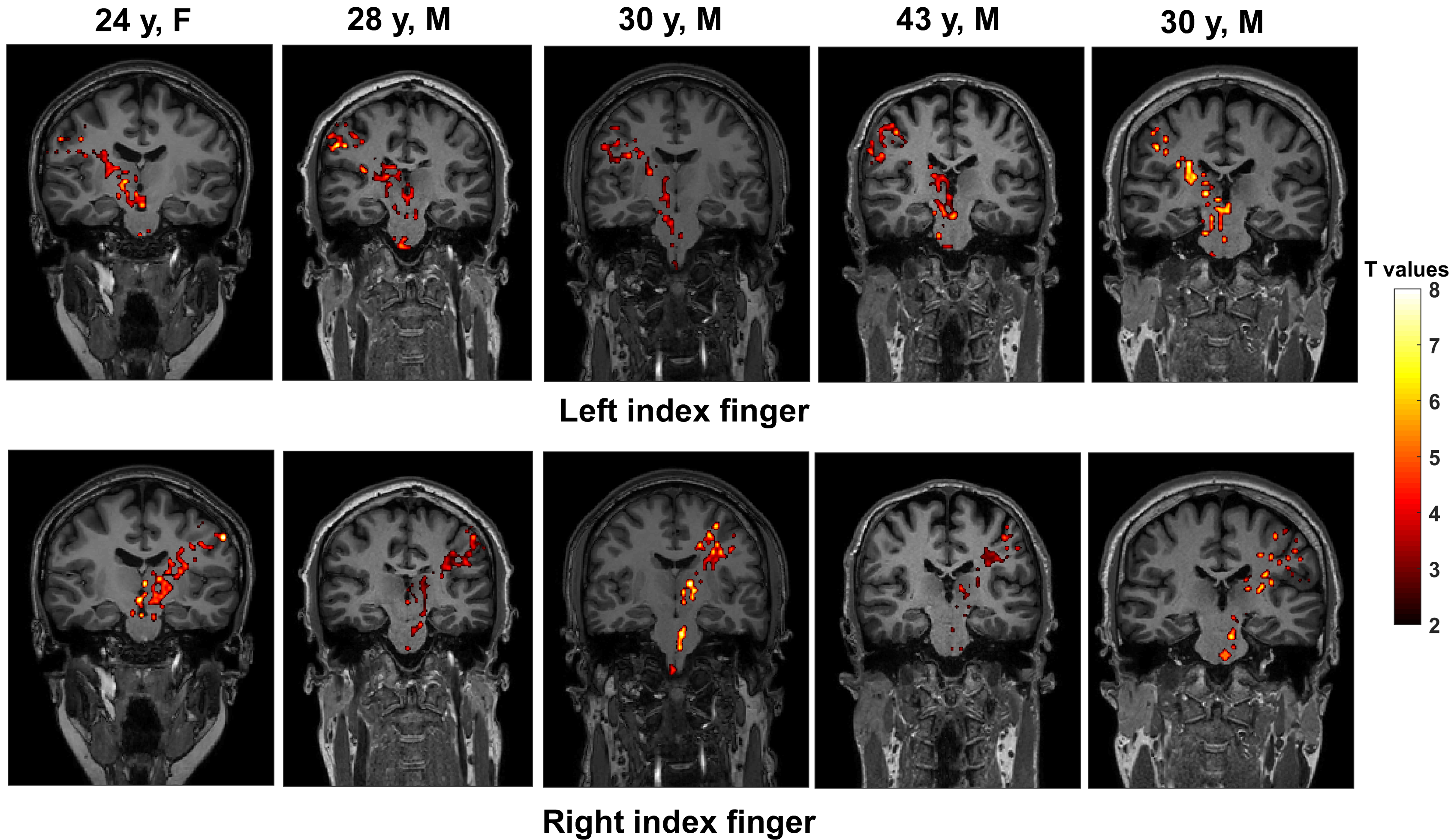

Supplement: Supplementary file 5 — Supplementary file5 (TIF 2674 KB) [file 13246_2024_1484_MOESM5_ESM.tif]

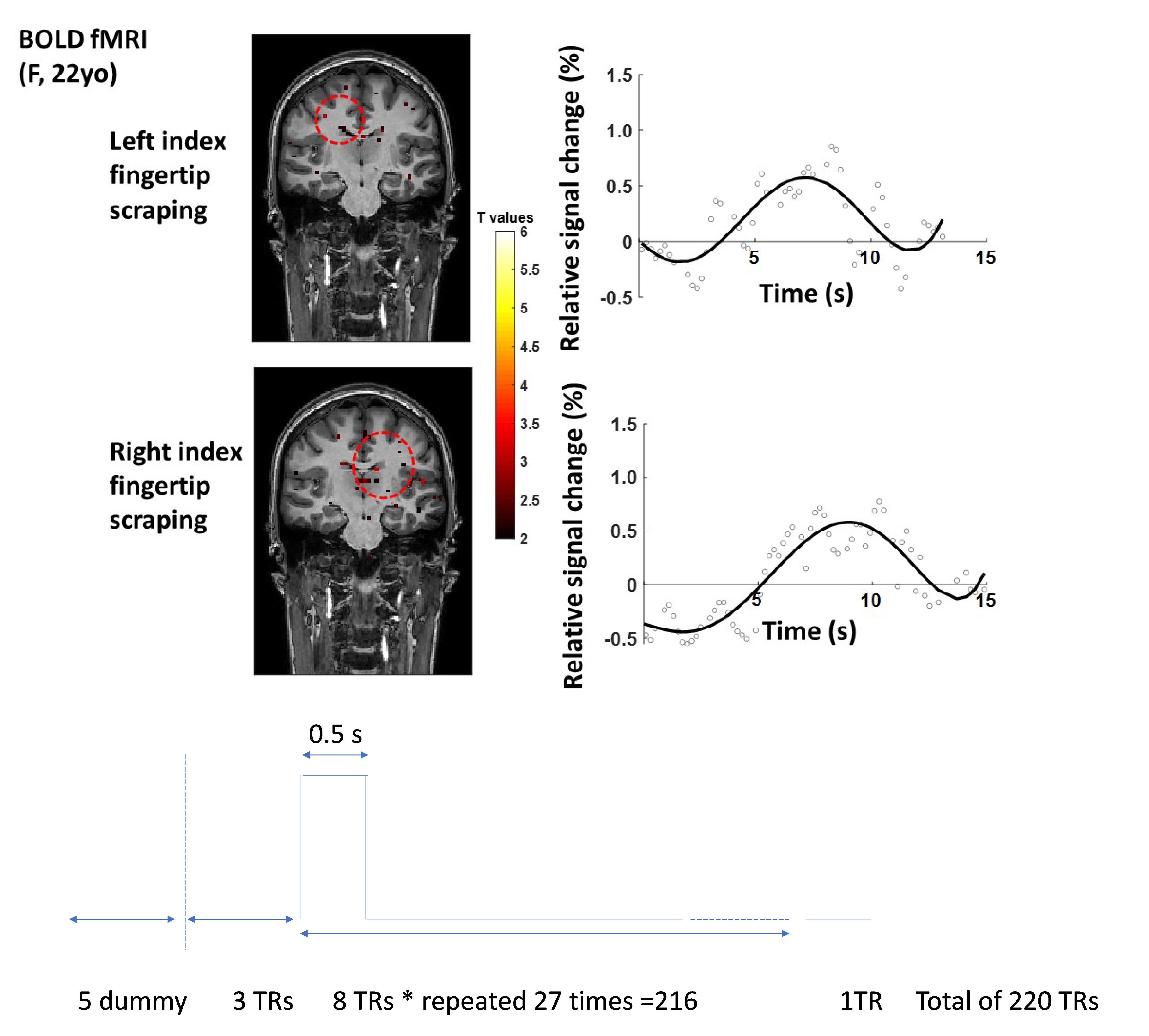

Supplement: Supplementary file 6 — Supplementary file6 (JPG 994 KB) [file 13246_2024_1484_MOESM6_ESM.jpg]
