# Supplementary material for: Functional conductivity imaging: quantitative mapping of brain activity
Source: Phys Eng Sci Med. 2024 Sep 11;47(4):1723–38. doi: 10.1007/s13246-024-01484-z (PMC11666624; doi:10.1007/s13246-024-01484-z)
Supplement: Supplementary file 7 — Supplementary file7 (DOCX 16 KB) [file 13246_2024_1484_MOESM7_ESM.docx]

**Supplementary figures**

**Supplementary Figure 1. Axial conductivity maps showing additional slices above and below slice #166 featured in Figure 2**. Conductivity images were processed in MATLAB R2018b (The MathWorks Inc, Natick, Massachusetts, USA) using a first level (general linear model) GLM and convolving the signal using the derived conductivity response function. The obtained T-value maps were co-registered to the high-resolution T1W images in SPM 12 (The Welcome Centre for Human Neuroimaging, UCL, London, UK). Histogram smoothing was applied during estimation and reslice using 4^th^ degree B-Spline interpolation. The co-registered T-values maps were overlayed on the T1W images for anatomical reference. Conductivity response functions were estimated by fitting Laguerre polynomials using Numpy (1.21.0).

**Supplementary Figure 2**. **Consistency of responses to visual stimulation.** Tissue conductivity response functions to 0.5s of 8 Hz flashing chequerboard measured in the same two participants at the same time of day on two different days. Repeat measurements were obtained on the same day in one of the participants. Data were fitted in using Laguerre polynomials in NumPy (1.21.0) to obtain the conductivity response functions shown. Agreement between response functions for each tissue was estimated using intraclass correlation coefficient in MedCalc for Windows, version 19.4 (MedCalc Software, Ostend, Belgium).

**Supplementary Figure 3 Phase stability.**

A phantom (ADNI) was scanned using bFFE (TR/TE=1.90/0.78 ms, 3mm iso, flip angle 25°, CS factor 4, dynamic scan duration 1.94 s, 5 dummy scans and 220 dynamic scans). After detrending, global phase correction was applied by using a simple spatial smoothing (Gaussian), as reported. The magnitude (A) and phase images (B) from the the 10^th^ and 11^th^ dynamics of the phantom are shown. The red circles show the areas used to calculate image SNR_0_, and the time courses from the blue voxel are shown in C and D. Phase stability was calculated, including the SD of corrected phase, 1/SNR_0_ (SNR_0_: the image SNR), and 1/tSNR (tSNR: temporal SNR of image dynamics). To examine the SD of phase, a global phase correction was used to alleviate scan-to-scan variation. Although there are no phase correction methods investigated for bFFE, spatial smoothing was found to reduce scan-to-scan variation in EPI (Hagberg 2012). The measures of phase stability as described above, were 1/SNR_0_ = 0.0064 rad (0.365°), 1/tSNR = 0.0081 rad (0.462°), SD of corrected phase = 0.0048 rad (0.275°). The first two measures had slightly larger values as expected as they were calculated from raw magnitude images. Overall, the phase stability was < 0.5°. This agrees with the values reported by Schmidt [36].

Phase stability in vivo also reflects natural jitter in the MREPT signal (similar to that seen in “resting state” fMRI) so is larger than that seen in a phantom.

**Supplementary Figure 4. Figure 6 tissue masks.** A. Masks for experiment shown in Fig,6A, B, Masks for experiment shown in Fig. 6B.

**Supplementary Figure 5 Consistent tissue conductivity changes in response to finger stimulation in five different individuals.** Single coronal slices from image pack showing significant changes in tissue conductivity in response to scraping the left or right index finger for 0.5s with a plastic fork. Conductivity images were processed in MATLAB R2018b (The MathWorks Inc, Natick, Massachusetts, USA) using a first level (general linear model) GLM.

**Supplementary Figure 6 BOLD response to 0.5s scraping of the left or right index fingers.** Coronal slice showing functional (BOLD) activation in response to 0.5 s stimulation of the left or right index fingers following 0.5s scraping with a plastic fork. Acquired at 3T using single-shot echo-planar imaging, SENSE = 2.9, multiband 3, TR/TE = 2000/30 ms, FOV 240 x 240, 3mm isotropic resolution with 5 dummy scans and 220 dynamics. The experimental paradigm is shown below. This experiment was performed at the request of a reviewer and is included to illustrate the relative insensitivity of BOLD fMRI to the small stimulus used in the equivalent funCI experiment.

**References**

Hagberg, G. E.; Bianciardi, M.; Brainovich, V.; Cassara, A. M.; Maraviglia, B., Phase stability in fMRI time series: effect of noise regression, off-resonance correction and spatial filtering techniques. *Neuroimage* **2012,** 59, (4), 3748-3761.
